# Various anthracyclines exhibit differential cytotoxic effects related to CBR1-induced resistance in lung cancer cells

**DOI:** 10.1007/s12032-025-02893-0

**Published:** 2025-07-08

**Authors:** Kamil Piska, Paulina Koczurkiewicz-Adamczyk, Paweł Kochanowski, Sylwia Bobis-Wozowicz, Benedykt Władyka, Elżbieta Pękala

**Affiliations:** 1 Department of Pharmaceutical Biochemistry, Faculty of Pharmacy, Jagiellonian University Medical College, Medyczna 9 St, Krakow, Poland; 2 Doctoral School of Exact and Natural Sciences, Jagiellonian University, Krakow, Poland; 3 Malopolska Centre of Biotechnology, Jagiellonian University, Krakow, Poland; 4 Department of Cell Biology, Faculty of Biochemistry, Biophysics and Biotechnology, Jagiellonian University, Krakow, Poland; 5 Department of Analytical Biochemistry, Faculty of Biochemistry, Biophysics and Biotechnology, Jagiellonian University, Krakow, Poland

**Keywords:** Multidrug resistance, Biotransformation, Anthracyclines, Non-small-cell lung cancer

## Abstract

Anthracyclines are widely used anticancer agents with a complex mechanism of action involving topoisomerase II inhibition and DNA intercalation. Despite their clinical efficacy, their use is limited by cancer cell resistance, linked to the formation of secondary alcohol metabolites via carbonyl reductase 1 (CBR1)-mediated reduction. These metabolites exhibit reduced anticancer activity, positioning CBR1 as significant factor in determining therapy outcomes. The study aimed to elucidate the role of CBR1 in mediating the differential responses of various anthracyclines in cancer cells. The role of CBR1 in cancer resistance against five anthracyclines: doxorubicin, daunorubicin, epirubicin, idarubicin, and aclarubicin, was examined in A549 lung cancer cells transduced with the CBR1. Anthracyclines were found to present significant differences in activity related to CBR1 overexpression. Surprisingly, aclarubicin was the most dependent on CBR1 among the tested compounds, while it exhibited a low reaction velocity when catalyzed by recombinant CBR1. The findings reveal critical differences in anthracycline susceptibility to CBR1, offering insights into resistance mechanisms.

## Introduction

Anthracyclines are a group of anticancer agents widely used in clinical oncology. Doxorubicin (DOX) and daunorubicin (DAUN) were isolated in the 1960s from Streptomyces peucetius. Their mechanisms of action and associated toxicities remain important subjects of pharmacological research. The primary mechanisms of anthracycline action involve the inhibition of topoisomerase II and DNA intercalation. However, their effects on cellular biology are far more complex, involving interactions with multiple molecular targets that culminate in cytotoxic and apoptotic effects [1, 2].

Despite their potent anticancer clinical efficacy, the application of anthracyclines is limited by two major challenges: the development of cancer cell resistance and cardiotoxicity. Resistance reduces the therapeutic response, while cardiotoxicity, a severe adverse drug reaction, can lead to arrhythmias, myocarditis, dilated cardiomyopathy, and ultimately, congestive heart failure [3].

Both resistance and cardiotoxicity have been linked to anthracycline metabolism. A key process is the two-electron reduction of the side chain carbonyl group, which produces secondary alcohol metabolites. These metabolites lack the strong anticancer properties of their parent compounds and exhibit significantly higher cardiotoxic potential [4]. This metabolic pathway has been documented for several anthracyclines, including DOX, DAUN, epirubicin (EPI), and idarubicin (IDA). This reduction is catalyzed by cytosolic enzymes, including carbonyl and aldo–keto reductases. Among them, carbonyl reductase 1 (CBR1) is one of the most extensively studied enzymes [5, 6].

CBR1 expression levels in tissues have been shown to influence cancer cell sensitivity, drug pharmacokinetics, and the risk of cardiotoxicity [5, 7, 8]. Furthermore, polymorphisms in the *CBR1* gene have been identified as factors affecting therapeutic outcomes [9]. Inhibition of CBR1 by compounds such as curcumin, piperlongumine, and flavonoids has demonstrated a significant chemosensitizing effect against anthracyclines, highlighting CBR1 as a potential drug target [10–12]. However, despite substantial research on CBR1-dependent resistance, comprehensive data comparing the susceptibility of different anthracyclines to this detoxification mechanism is lacking. Most studies have focused on DOX and DAUN due to their widespread clinical use, rather than on a pharmacological rationale.

In this study, an effort was made to fill gaps in knowledge regarding differences in anthracycline susceptibility to the CBR1-dependent mechanism of cancer cell resistance. To address this, five clinically relevant or experimental anthracyclines: DOX, DAUN, EPI, IDA, and aclarubicin (ACLA) were investigated with respect to CBR1-mediated resistance in cancer cells. It is assumed that structurally diverse anthracyclines, characterized by varying affinities for CBR1 and nuanced differences in their mechanisms of action, will exhibit differential sensitivity to CBR1-mediated resistance. For this purpose A549 non-small-cell lung cancer cells were transduced with the *CBR1* gene via lentivirus, resulting in stable enzyme expression. A549 cell line was chosen because it exhibits low native expression of CBR1 and is relevant to anthracycline-based lung cancer therapy. To determine whether ACLA serves as a substrate for CBR1, reactions with the recombinant enzyme were also conducted.

## Materials and methods

### Cell culture

A549 non-small-cell lung cancer cell line (ATCC, CCL-185) and its transduced counterparts were used in this study. Cells were cultured in standard conditions (37 ℃, 5% CO_2_) in DMEM supplemented with 10% FBS and 1% antibiotics (Gibco).

### Lentiviral vectors production and cells transduction

A549/CBR1 cell line overexpressing CBR1 and A549/ev (empty vector) control cells were generated by lentiviral transduction. Lentiviral vector particles were produced by transient transfection of HEK293T/17 packaging cell line (ATCC, CRL-11268) with CBR1 expression vector (LV 108186; abm; Richmond, BC. Canada) along with psPAX2 and pMD2G packaging plasmids (Addgene #12,260 and #12,259, respectively), using Lipofectamine2000 (Invitrogen) as a transfection agent. Subsequently, cell supernatant was filtered through 0.2 mm pores PVDF filters (Millipore) and stored at − 80 ℃. Vector titration was performed using HEK293T cells and measurement of EGFP expression by flow cytometry (BD LSR II; BD Biosciences). A549 cells were transduced with the lentiviral particles in DMEM medium in 12-well plates at cells density of 5 × 10^4^ with addition of 10 μg/ml of polybrene (Milipore) by 24 h incubation at 37 ℃ in a cell incubator. After 3 days of expansion, the EGFP-positive cells were sorted using the FACS Aria III cell sorter (BD Biosciences). Procedure of cells multiplication and sorting was performed 3 times. Procedure resulted in establishment of A549/CBR1 cell line. A549/ev cell line was established in an analogous way; however, it was transduced with empty vector (the vector without *CBR1* gene).

### Western blot

A549/CBR1 and A549/ev cells were seeded in 58 cm^2^ culture dishes and cultured at 37 ℃ until 90% confluence was reached. Next, cells were washed with cold PBS and lysis buffer (165 mM Tris, 150 mM NaCl, 0.3 mM sodium azide, 1% Triton X, proteases inhibitor) was added. Lysates were homogenized and centrifuged (10 min, 1.5 × 10^4^ g, 4 ℃). Protein concentration was measured with Bradford method. Samples were diluted with buffer containing dithiothreitol (23 mg/ml), incubated at 95 ℃ for 5 min and volumes equivalent for 30 μg of protein were transferred to wells in polyacrylamide gel, where electrophoresis separation was performed (200 V, 45 min). Next, proteins were transferred to PVDF membrane (100 V, 350 mA, 60 min). Membranes were washed in TBST (10 mM Tris–HCl, 150 mM NaCl, 0.05% Tween-20), and 5% milk in TBST solution. Membranes were incubated with 1:500 diluted anti-CBR1 rabbit IgG antibodies (Invitrogen, # MA5-37,892) in 3% BSA solution for 18 h, and then with 1:3000 secondary goat antibody anti-rabbit IgG conjugated with horseradish peroxidase (Invitrogen, #656,120). Membranes were washed and incubated with peroxidase substrate (Super Signal West Pico, Invitrogen), and the chemiluminescence was read in C-DiGit (Li-cor) blot scanner.

### Cells viability assay

#### MTT

Cells were seeded at a density of 10^4^ per well in 96-multiwell plates in 200 μl of medium. After 24 h, solutions of anthracyclines were added in a broad concentration range. After next 48 h, 10 μl of MTT solution (5 mg/ml; Sigma Aldrich) was added to each well. Then, after 3-h incubation, as formazan crystals appeared in the bottom of wells, medium was removed and 100 μl of DMSO was added to dissolve formazan. Absorbance of the solution was measured at 570 nm on a plate reader (Spectra Max iD3, Molecular Devices). Viability was calculated by dividing average absorbance of each experimental condition by the absorbance of control, multiplied by 100 (%). Three technical repeats were used. IC_50_ of each anthracycline was determined from plot Log(concentration) vs. viability using GraphPadPrism 6. Average IC_50_ was calculated from three separate experiments.

#### The Sulforhodamine B (SRB) assay

Cells were seeded at a density of 10^4^ per well in 96-multiwell plates in 200 μl of medium. After 24 h, solutions of anthracyclines were added in a broad concentration range. After next 48 h, cells were fixed with trichloroacetic acid (50% w/v) for 1 h in 4 ℃. Cells were washed with water, and stained for 30 min with sulforhodamine B solution (0.4% in 1% acetic acid). Then cells were washed four times with 1% acetic acid, and the incorporated stain was solubilized in 10 mM Tris solution. Absorbance of the solution was measured at 565 on a plate reader (Spectra Max iD3, Molecular Devices). Viability was calculated by dividing average absorbance of each experimental condition by absorbance of control, multiplied by 100 (%). Three technical repeats were used. IC_50_ of each anthracycline was determined from plot Log(concentration) vs. viability using GraphPadPrism 6. Average IC_50_ was calculated from three separate experiments.

#### Recombinant CBR1 activity assay

Expression and the purification of the hCBR1 protein was performed as previously described [13]. A reaction mixture contained 0.1 M phosphate buffer (pH 7.4), recombinant CBR1 enzyme (0.5 μM) and reference (menadione, MEN 129 μM, equal to Km; or DAUN, 100 μM) or potential (ACLA, 100 μM) CBR1 substrates. 200 μM NADPH was added to initiate the reactions, and NADPH oxidation process was determined by measuring the decrease in absorbance at 340 nm (SpectraMax® iD3, Molecular Devices) through 10 min. Velocity of the reaction was calculated in OriginPro, by a linear regression method. Three separate experiments were conducted.

## Results

### Cells transduction

**Fig. 1:**
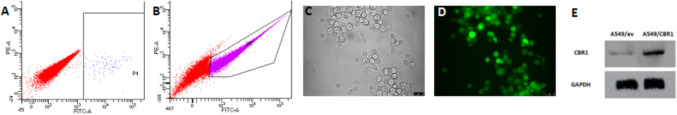
**AB** FACS plot of A549/CBR1 cells transduced with lentivirus during first (**A**) and second (**B**) sort. **CD** Images of A549/CBR1 cells suspension taken by Leica DMiL LED Fluo in visible (**C**) light and fluorescence (**D**) mode. **E** CBR1 protein level in A549/ev and A549/CBR1 cells

To establish cell line overexpressing CBR1, A549 non-small-cell lung cancer cells were transduced using a lentiviral vector. Green fluorescent protein (GFP) was used as a marker for transduced cells. Following transduction and selection of cells using a fluorescence-activated cell sorter, a population of cells exhibiting stable, high expression of the introduced genes (A549/CBR1) was isolated. Similarly, a control cell line (A549/ev) was established by transduction with a control vector lacking the CBR1 gene. An elevated level of CBR1 protein in A549/CBR1 cells compared to A549/ev cells was confirmed by Western blot analysis (Fig. 1). The obtained cells were utilized for experiments aimed at assessing the impact of CBR1 on the biological activity of anthracyclines.

### Anthracyclines cytotoxicity in CBR1-transduced and native cancer cells

Cells used in the study showed different sensitivity for anthracyclines treatment. In A549/ev cells, the most potent anthracycline was IDA followed by ACLA. DOX, EPI, and DAUN exhibited lower activity. In A549/CBR1, meaningful decrease of sensitivity against DAUN (2.05-fold), IDA (2.86-fold), and ACLA (24.56) was found, while no significant change of anthracyclines cytotoxicity was found in the case of DOX and EPI (Table 1).

**Table 1:** IC_50_ of anthracyclines were determined in three independent analysis (MTT assays)

Anthracycline	IC_50_ [µM]		Fold change (A549/CBR1)/(A549/ev)
	A549/ev	A549/CBR1	
DOX	0.336 ± 0.213	0.527 ± 0.151	1.57
EPI	0.553 ± 0.196	0.683 ± 0.167	1.23
DAUN	0.230 ± 0.133	0.471 ± 0.198	2.05^#^
IDA	0.033 ± 0.012	0.094 ± 0.021	2.86*
ACLA	0.073 ± 0.105	1.785 ± 0.988	24.56*

Average values and standard deviation are presented. Statistically significant difference in t-Student test * p < 0.05, # p < 0.1

**Fig. 2:**
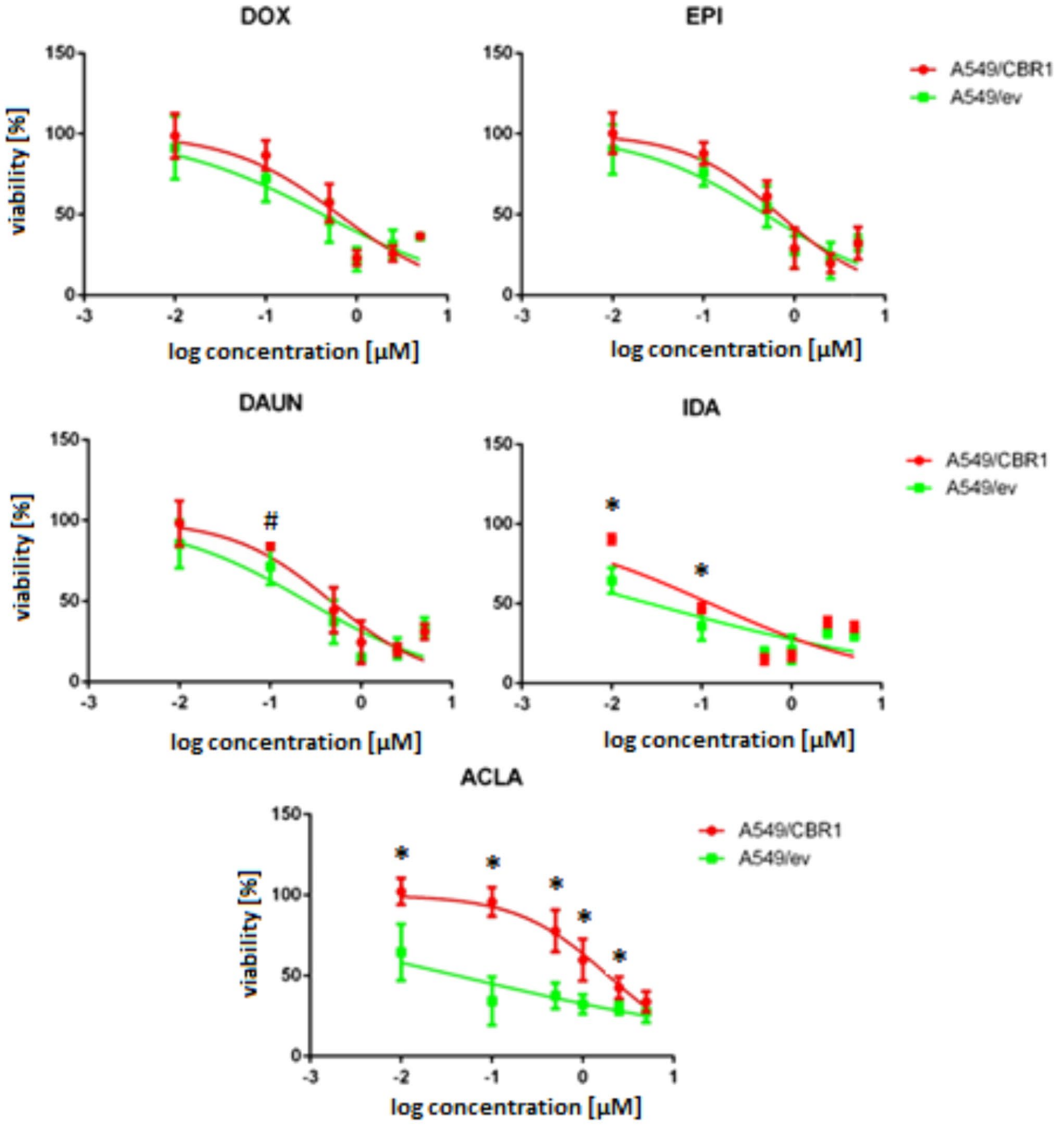
Influence of anthracyclines on cells viability in MTT assay. Figure represents viability of A549/ev and A549/CBR1 cells treated with anthracyclines for 48 h. Values on x-axis show drugs concentration in μM. Experiments were repeated three times, and statistical significance of differences was assessed using ANOVA with Tukey’s post hoc test (*p < 0.05, # *p* < 0.1)

In more detailed analysis of anthracyclines cytotoxicity, the most significant differences between A549/ev and A549/CBR1 sensitivity were found in concentrations near IC_50._ In very low, non-toxic submicromolar and very high concentrations, there were no differences (Fig. 2). IC_50_ represents a critical threshold where cells are most responsive to external modulation. At lower concentrations, drug-induced stress may be insufficient to elicit significant changes, while at higher concentrations, maximal cytotoxicity can obscure subtle effects. Thus, IC₅₀-level exposure provides an optimal window to observe differential responses related to CBR1 activity.

**Fig. 3:**
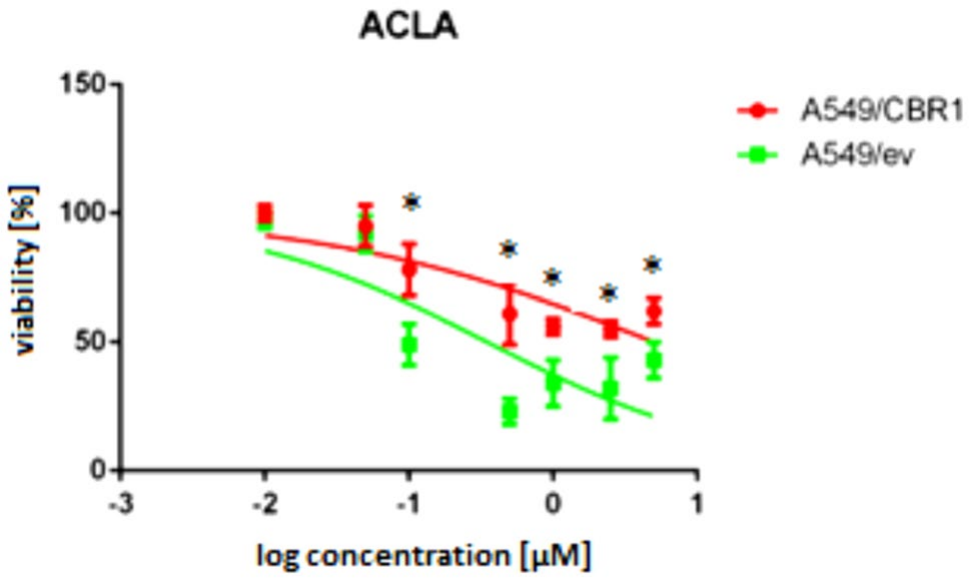
The effect of aclarubicin (ACLA) on the viability of A549/CBR1 and A549/ev cell lines after 48-h incubation in the Sulforhodamine B (SRB) assay. The graph represents the mean cell viability expressed as % ± SD. Experiments were repeated three times, and statistical significance of differences was assessed using ANOVA with Tukey’s post hoc test (*p < 0.05)

To confirm that very high effect of CBR1 overexpression on ACLA is not related to interference with MTT assay, SRB assay was performed. In SRB assay, similar level of cytotoxicity reduction was observed (Fig. 3). The IC_50_ value increased 14.88-fold. (Table 2).

**Table 2:** IC_50_ of ACLA was determined in three separate SRB assays

Anthracycline	IC_50_ [µM]		Fold change (A549/CBR1)/ (A549/ev)
	A549/ev	A549/CBR1	
ACLA	0.351 ± 0.113	5.226 ± 0.951	14.88*

Average values and standard deviation are presented. Statistically significant difference against native cell line in t-Student test * *p* < 0.05

### Reaction with CBR1 recombinant enzyme

**Fig. 4:**
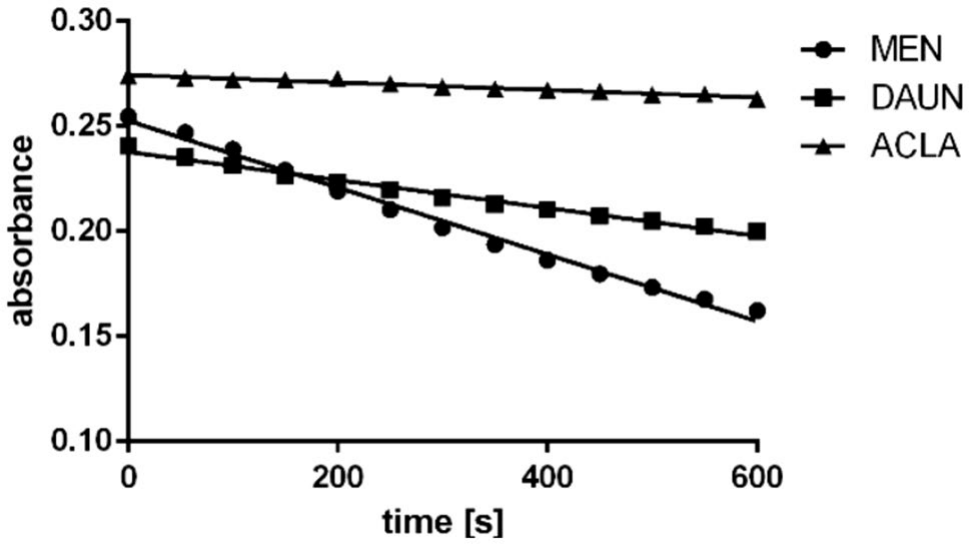
Decrease of absorbance at 340 nm (related to NADPH oxidation) with substrates with recombinant CBR1

To investigate if ACLA is a substrate for a reaction catalyzed by CBR1 enzyme, this anthracycline was incubated with recombinant CBR1. As control agents, known substrates were used: MEN, which is standard substrate usually used in inhibition studies, and DAUN, an anthracycline which is also known to be substrate for CBR1-mediated two-electron reduction. MEN and DAUN incubations led to significant oxidation of NADPH. The process, as indicated by the decrease in absorbance at 340 nm, was only mildly affected by ACLA when applied at a high concentration equal to that of DAUN (Fig. 4). This is also confirmed by the velocities of the reactions, indicating very low velocity of the reactions for ACLA (Table 3).

**Table 3:** Velocities of the reactions catalyzed by CBR1 with different substrates

Substrate	Velocity of reaction [µM s^−1^]
MEN	0.12492
DAUN	0.05538
ACLA	0.01301

## Conclusions

Anthracyclines constitute a key class of chemotherapeutic agents widely used in oncology. Their continued relevance in clinical practice is partly attributed to advances in drug delivery systems. Nevertheless, cancer cells may develop acquired or present intrinsic resistance to these drugs. Over the years, numerous molecular mechanisms underlying anthracycline resistance have been elucidated. Recently, there has been a growing focus on the role of anthracycline-reducing enzymes, such as carbonyl reductase 1 (CBR1), as contributors to drug resistance [14]. The literature contains many reports on the influence of individual enzymes or their inhibitors on anthracycline activity, although these typically focus on a single anthracycline. In this study, five clinically used and experimental anthracyclines were compared in terms of their CBR1-dependent resistance.

In this study, a cellular model was developed to investigate the role of CBR1 in cancer cell resistance. Cells were transduced using a lentiviral vector, resulting in stable expression of the enzyme in cells. A549 non-small-cell lung cancer cell line was chosen, because anthracyclines are used in clinical oncology in a treatment of this type of cancer or were investigated in clinical trials [2, 15, 16]. Native cells were found to exhibit low level of CBR1 expression. Moreover, A549 cells were previously widely utilized in studies related to CBR1 and anthracyclines resistance [13, 17, 18]. 

In A549/ev cells, DOX, EPI, and DAUN exhibited similar, relatively low levels of cytotoxicity. IDA and ACLA were more active, with IC_50_ values below 100 nM. Elevated CBR1 expression in A549/CBR1 cells was associated with altered sensitivity to various anthracyclines.

The effect on DOX and EPI activity was weak and statistically insignificant, with IC_50_ values increasing by 1.53- and 1.27-fold, respectively. Over twofold increase of IC_50_ was found in case of DAUN (*p* < 0.1). Also, significant activity alteration associated with CBR1 level in cells was found for IDA. The IC_50_ of IDA increased from 0.033 μM in A549/ev cells to 0.094 μM in A549/CBR1 cells, representing a 2.86-fold increase (*p* < 0.05). The greatest difference was found for ACLA. In A549/ev cytotoxicity of ACLA was estimated for IC_50_ of 0.073 µM, while in A549/CBR1 cells, IC_50_ increased 24.56 times to a level of 1.785 μM. To confirm this very intense effect, and exclude interference experimental condition with MTT assay, additionally, SRB assay was performed. In this case, IC_50_ of ACLA was 0.351 μM in A549/ev cells and 5.226 μM in A549/CBR1 cells, which means almost 15-fold increase.

In previous studies, transfection of colon cancer cells (LoVo, DLD1) with *CBR1* also lead to mean increase of cell resistance against DOX. On contrary CBR1 knockout in MKN45 cell line was associated with increased cells sensitivity [8]. The effect appears to be a similarly weak desensitization.

Transfection of chronic myelogenous leukemia K562 cells with the *CBR1* gene caused a significant decrease in the cells’ sensitivity to DAUN. However, no such effect was observed with DOX [19], which is consistent with our observation of a weak effect of CBR1 on DOX activity, in contrast to the moderate alteration of DAUN activity in A549 cells.

Higher dependence of DAUN than DOX on CBR1 level is also supported by kinetics studies, indicating that DAUN is more dependent for CBR1-dependent catalysis than DOX [6, 20].

High dependence of cells resistance of IDA, and low dependence of EPI on CBR1 activity, is in accordance with kinetics data. IDA was found to be a substrate with high affinity to CBR1, while catalytic activity of CBR1 toward EPI seems to be low [6, 21].

The most surprising is the influence of CBR1 on ACLA activity. While CBR1 catalytic activity is believed to affect anthracyclines, through reduction of carbonyl moiety, ACLA has carbonyl carbon which is, however, the part of an amide moiety. Therefore, it is not substrate of reaction catalyzed by CBR1 and cannot be reduced. In studies over metabolism, reduction of carbonyl moiety of sugar side chain was observed. Molecular modeling studies indicated lack of a possibility of ACLA to bind with CBR1 [6]. Additionally, experiment with recombinant CBR1 was performed, indicating very low velocity of the reaction catalyzed by CBR1 in high ACLA concentration. However, CBR1 highly increased resistance of cells against ACLA. This may be associated with other properties of the enzyme unrelated to biotransformation process of two-electron side chain reduction. The enzyme could cause resistance by antioxidant activity, decreasing ROS-induced damage in cells. Decreasing ROS-induced damage by CBR1 was observed in cells treated with arsenic trioxide [7] and cisplatin [22]. Since ROS generation may be important mechanism of ACLA anticancer activity [23], CBR1 may influence its activity by this mechanism. There are also reports on the association of CBR1 with hypoxia [22], autophagy, and ferroptosis [24]; therefore, there are also other mechanisms which may involve in A549 cells resistance induced by CBR1 overexpression.

Currently, ACLA is recognized as an anthracycline which does not undergo cross resistance with other anthracyclines. Cancer cells resistant to other anthracyclines based on the ABCB1 overexpression mechanism is believed to maintain sensitivity against ACLA [25–27]. In this study, novel resistance mechanism of cancer cells against ACLA was proposed; therefore, CBR1 expression level and activity should be considered in studies investigating ACLA anticancer activity.

The results of this study may have relevance for future research in clinical oncology. Variations in CBR1 expression and known genetic polymorphisms have been shown to influence therapy outcomes, making this research valuable for understanding patient-specific responses to different anthracyclines [28, 29]. While the anticancer activity of DOX and EPI is expected to be independent of CBR1 status, the efficacy of DAUN, IDA, and particularly ACLA may be reduced in patients whose tumors exhibit CBR1 overexpression or specific polymorphic variants. Our findings indicate that CBR1 expression may modulate cellular sensitivity to ACLA, suggesting a potential role in resistance mechanisms. This could have clinical implications, as elevated CBR1 levels might partially explain reduced therapeutic responses to ACLA in certain cases. Although our study was conducted in vitro, these observations highlight the need for further clinical investigations to assess whether CBR1 expression correlates with treatment outcomes and whether it could serve as a predictive marker or therapeutic target to improve the efficacy of ACLA-based regimens.

Moreover, the results may inform the development of next-generation anthracyclines with decreased affinity for CBR1, hence, improved efficacy and safety profiles. It is also essential for search of CBR1 inhibitors, because results suggest activity of which anthracyclines may be modulated with use of inhibitors.

## Data Availability

No datasets were generated or analyzed during the current study.
